# Evaluation of Essential and Toxic Elements in the Blood of 0–14-Year-Old Children in Hunan, China From 2013 to 2019: A Retrospective Analysis

**DOI:** 10.3389/fpubh.2022.739880

**Published:** 2022-04-18

**Authors:** Shan Tan, Yang Yang, Zhiheng Chen, Lingling Zhao, Zuocheng Yang, Hongmei Dai, Wei He, Mei Jiang, Yanhua Yao, Ke Huang, Liu Li, Pengfei Zhu, Shasha Xu, Mingyi Zhao, Minghua Yang

**Affiliations:** Department of Pediatrics, The Third Xiangya Hospital, Central South University, Changsha, China

**Keywords:** essential elements, toxic elements, children, Hunan, nutrition

## Abstract

**Objective:**

The aim was to investigate the distribution and correlation of Ca, Mg, Zn, Cu, Fe, Pb, and Cd in the blood of children aged 0–14 years in Hunan, China, which may serve to provide a basis for clinical guidance on child health.

**Study Design:**

A retrospective analysis was carried out. Concentrations of all elements were determined by atomic absorption spectrophotometry. Distributions were analyzed and compared among different age, sex, and year groups by the Kruskal–Wallis test, the chi-square test, and the Fisher's exact test. Spearman's rank correlation coefficient was used to evaluate the association between every pair of elements.

**Results:**

A total of 46,951 children were involved in this study from 2013 to 2019. The median blood levels of elements were 13.51 μmol/L (Cu), 58.69 μmol/L (Zn), 1.70 mmol/L (Ca), 1.40 mmol/L (Mg), 7.46 mmol/L (Fe), 35.00 μg/L (Pb), and 1.00 μg/L (Cd). Girls had a higher level of Ca and lower levels of Pb and Cd than boys. Cu and Ca showed an upward trend, and Mg and Pb showed a downward trend by year. Zn and Fe increased and Ca decreased significantly with age. The deficiency rates of Fe and Zn decreased significantly by year, while Ca and Cu increased significantly by year. Cd exposure in this area was relatively low.

**Conclusion:**

Most children had normal levels of the essential elements Ca, Cu, and Mg and the toxic elements Pb and Cd. Severe deficiencies in Zn and Fe were observed in the relatively younger children but improved with age. Persistent efforts in reducing Pb exposure might still be needed.

## Background

Childhood is in the most critical period of physical and intellectual development; it is characterized by a large demand for nutrition and low tolerance to the deficiency of trace elements and excess of toxic metal elements (1). The usual essential elements, such as calcium (Ca), magnesium (Mg), zinc (Zn), copper (Cu), and iron (Fe), although the body requires very small amounts, are of great significance for human health because of their function in physical and intellectual development, oxygen transport, haematopoietic function, immunologic function, and biological metabolism (2–4). Zn is thought to be a vital substance for neuroprotection (5) and myelination (6), and the latter is also considered a copper-dependent process (7). A corresponding study also confirmed that children with cerebral palsy were characterized by significantly lower Cu and Zn by 6 and 8%, respectively (8). Iron, as a blood constituent, participates in the synthesis of hemoglobin, cytochromes, and other proteins or enzymes and is indispensable for respiration, mitochondrial function, and energy production (9). Iron deficiency is one of the most severe health problems worldwide and particularly influences children, leading to anemia as the main consequence (10). Many proteins, such as troponin C and actin, possess the same binding sites for Ca and Mg, which results in competition between these two elements for sites (11). Ca is a constituent of teeth and bones and is essential to control membrane potential and promote coagulation (11). By blocking the release of Ca, Mg causes smooth muscle relaxation, and intravenous magnesium sulfate is used in children with asthma (12). In addition, Mg is an indispensable cofactor for glucose in cells, whose deficiency impairs the cellular ability to resist oxidative damage and leads to diabetes exacerbation (13).

Cadmium (Cd) and lead (Pb) are non-essential toxic heavy metals widely distributed in the environment. The International Agency for Research on Cancer (IARC) classified Cd and Pb as human carcinogens (Group I), and Pb poisoning and Cd poisoning are now recognized as a prominent public health issue. It was shown that Cd increased the risk of cancer and mortality and caused kidney damage and a decline in bone mineral density, which gave rise to diseases such as osteomalacia and osteoporosis (14, 15). The adverse effects of Pb on the human body present on the nervous system and endocrine system, mainly manifesting as dyslexia, intellectual decline, attention deficit hyperactivity disorder (ADHD), and other mental behavior problems (16–18). There is no safe level of Pb and Cd exposure in children. Low blood levels of Cd may be associated with adverse neurodevelopmental outcomes or neurobehavioral performance (19, 20). Additionally, very low blood Pb levels were associated with intellectual deficits (21–24). Recognizing the potentially harmful effect of Pb on health, especially in children who are more susceptible to Pb exposure than adults, the United States Centers for Disease Control and Prevention (CDC) lowered the Pb poisoning concentration from 100 μg/L to 50 μg/L in 2012 (25). As a result of banning the use of leaded gasoline since 2000, Pb exposure in China has consistently fallen over the past decade. A project including 47,346 children aged 0–6 years from 11 cities all over China showed that the blood lead levels (BLLs) of Chinese children decreased from 46.38 μg/L in 2004 to 37.17 μg/L in 2013 (26). The relationships between these essential elements and toxic elements are controversial. It is important to highlight that the increase in BLLs will inevitably affect the absorption and utilization of Ca, Fe, Zn, Cu, and other essential elements, while the lack of these elements will promote the absorption of Pb, thereby increasing its toxic effects in the body (27, 28).

Given the critical period in which the children are developing, it is of great significance to understand the status of various essential and toxic elements in the blood in a timely and accurate manner to guide children on a balanced diet, to supply and adjust trace elements required by the body in time, and to maintain normal growth and development. In this study, atomic absorption spectrophotometry was used to investigate the distribution and correlation of essential and toxic elements (Ca, Mg, Zn, Cu, Fe, Pb, and Cd) in the blood of 0–14-year-old children from Hunan, China from 2013 to 2019. To the best of our knowledge, this is the first large sample study about changes in the concentrations of essential and toxic elements for the children's age and time trends in Hunan, China, with a view on providing a basis for clinical guidance of child health.

## Methods

### Sample Collection

A total of 46,951 children aged 0–14 years were involved in this retrospective study, including 26,429 boys and 20,522 girls. All the children and their parents were residents of Hunan Province, and the children underwent regular health examinations in the child health care department of the Third Xiangya Hospital from January 1, 2013, to December 31, 2019. Participants who were complicated with (1) acute severe liver or kidney dysfunction; (2) ARDS or other communicable diseases; (3) schizophrenia, severe bipolar disorder and other neurodegenerative diseases; (4) leukemia, lymphoma, and other cancers; (5) congenital malformation; (6) severe inherited diseases; and (7) a history of transfusion or operations were excluded from this study. This study was approved by the Ethics Committee of the Third Xiangya Hospital.

### Sample Preparation

Blood samples were collected in the child health center and then analyzed in the pediatric laboratory of The Third Xiangya Hospital of Central South University. For all the participants, ~2 ml of fasting venous blood was sampled using lithium heparin vacuum blood collection tubes specific for trace elements, followed by gentle mixing. The mouths of the tubes were sealed tightly to prevent leakage when submitted for examination. If the blood sample could not be submitted in time, it was refrigerated, stored at 2–8°C, and the examination was finished within 72 h. The samples were mixed completely prior to the examination, and 40-μL blood was extracted by micropipette from every sample and placed into diluent specific for trace elements, followed by mixing completely and examination.

### Element Measurement

The concentrations of essential and toxic elements were measured by atomic absorption spectrophotometry. Cu, Zn, Ca, Fe, and Mg concentrations were detected using a BOHUI 7100s analyzer; Pb and Cd were detected using a BOHUI 2101s analyzer. Related reagents and calibrators were purchased from Bohui Innovation Technology Co., Ltd. (Beijing, China). The concentrations of all elements except for Cd can be accurate to 0.01. The concentration of Cd can be accurate to 1. Reference values in our laboratory were as follows: Cu: 11.8–39.3 μmol/L, Ca: 1.55–2.10 mmol/L, Mg: 1.12–2.06 mmol/L, Fe: 7.52–11.82 mmol/L, Pb: 0–100 μg/L, Cd: 0–5 μg/L, and Zn: (0–0.99 years) 58–100 μmol/L; (1–1.99 years) 62–110 μmol/L; (2–2.99 years) 66–120 μmol/L; (3–4.99 years) 72–130 μmol/L, and (≥5 years) 76.5–150 μmol/L. “Deficiency” was defined as values below the normal threshold. Intoxication levels were as follows: Pb: >100 μg/L and Cd: >5 μg/L. The Pb poisoning standard was revised by the U.S. Centers for Disease Control in 1991.

### Statistical Analysis

All statistical analyses were conducted using SPSS version 21.0. Quantitative data were expressed as quartiles, and categorical data were expressed as percentages. We first counted the concentrations of all elements in participants of different genders, ages, and years, and the latter two results were further used to draw straightforward figures, illustrating every element trend by age (0–14 years) and year (2013–2019) using R (version 4.0.3). Next, the participants were divided into four groups according to their ages: Group A: ~0 (age <1 year, *N* = 19,262); Group B: ~1 (1 ≤ age <3 years, *N* = 12,803); Group C: ~3 (3 ≤ age <6 years, *N* = 6,056); Group D: ~6–14 (6 ≤ age <14 years, *N* = 8,830). The Kruskal–Wallis test was used to compare the differences in element concentrations in two or more groups based on sex, age, and year. The mean values and standard deviations of all elements are also shown in Table 1.

**Table 1:** Comparison of toxic and essential element levels in the blood of children age from 0 to 14 in Hunan, China, based on gender, age, and year.

**Group**	***N***	**Cd (μg/L)**	**Cu (μmol/L)**	**Zn (μmol/L)**	**Ca (mmol/L)**	**Mg (mmol/L)**	**Fe (mmol/L)**	**Pb (μg/L)**
**Gender**
Boys	26,429	1.00 (0.00–1.00)	13.51 (12.33–15.98)	58.8 (49.30–68.88)	1.70 (1.65–1.75)	1.40 (1.32–1.52)	7.48 (6.99–7.98)	35.44 (25.00–47.00)
		0.80 ± 0.79	14.48 ± 3.54	59.60 ± 13.84	1.68 ± 0.17	1.43 ± 0.13	7.48 ± 0.75	36.05 ± 16.90
Girls	20,522	1.00 (0.00–1.00)^**^	13.52 (12.36–16.10)	58.31 (49.44–68.27)	1.70 (1.65–1.76)^**^	1.40 (1.32–1.52)	7.45 (6.98–7.95)	33.83 (24.00–45.43)^**^
		0.78 ± 0.90	14.54 ± 3.54	59.32 ± 13.53	1.69 ± 0.16	1.43 ± 0.13	7,46 ± 0.73	34.79 ± 16.07
**Age (years)**
A(0~)	19,262	1.00 (0.00–1.00)	13.66 (12.57–16.25)	50.16 (43.62–56.89)	1.73 (1.69–1.77)	1.39 (1.32–1.50)	7.12 (6.63–7.66)	32.05 (23.00–42.87)
		0.76 ± 0.75	14.78 ± 3.55	50.98 ± 10.33	1.72 ± 0.19	1.41 ± 0.12	7.16 ± 0.66	32.93 ± 15.07
B(1~)	12,803	1.00 (0.00–1.00)^a^	13.51 (12.33–16.02)^a^	57.36 (50.63–63.36)^a^	1.71 (1.67–1.76)^a^	1.42 (1.32–1.53)^a^	7.41 (7.01–7.90)^a^	36.00 (25.16–47.22)^a^
		0.81 ± 0.82	14.53 ± 3.58	57.60 ± 10.05	1.71 ± 0.15	1.44 ± 0.13	7.44 ± 0.62	36.47 ± 17.09
C(3~)	6,056	1.00 (0.00–1.00)^a^	13.32 (12.27–15.71)^a^^b^	65.23 (61.16–71.50) ^a^^b^	1.63 (1.60–1.68)^a^^b^	1.42 (1.32–1.53)^a^	7.71 (7.26–8.15)^a^^b^	37.33 (26.24–50.64)^a^^b^
		0.83 ± 1.16	14.27 ± 3.51	67.43 ± 10.03	1.65 ± 0.11	1.44 ± 0.13	7.74 ± 0.63	38.27 ± 17.03
D(6~14)	8,830	1.00 (0.00–1.00)^a^	13.29 (12.17–15.71)^a^^b^^c^^*^	73.76 (70.16–80.59)^a^^b^^c^	1.62 (1.55–1.68)^a^^b^^c^	1.42 (1.33–1.53) ^a^^b^	7.96 (7.62–8.40)^a^^b^^c^	36.96 (25.08–50.00)^a^^b^^c^^*^
		0.82 ± 0.78	14.05 ± 3.42	75.29 ± 0.73	1.62 ± 0.13	1.44 ± 0.13	8.00 ± 0.77	37.78 ± 17.71
**Year**
2013	7,319	1.00 (0.00–1.00)	13.61 (12.68–15.37)	60.49 (49.42–69.20)	1.68 (1.64–1.72)	1.51 (1.41–1.61)	7.41 (6.88–7.95)	36.00 (20.00–49.00)
		0.87 ± 1.08	14.06 ± 2.21	60.01 ± 14.83	1.68 ± 0.07	1.51 ± 0.13	7.44 ± 0.73	34.74 ± 20.04
2014	6,584	1.00 (1.00–1.00)^e*^	12.88 (12.15–13.61)^e^	60.71 (51.2–68.98)	1.68 (1.62–1.72)^e^	1.43 (1.33–1.55)^e^	7.40 (6.87–8.03)	36.00 (26.00–51.00)^e^
		0.89 ± 0.64	12.90 ± 1.76	60.24 ± 13.67	1.67 ± 0.08	1.45 ± 0.13	7.47 ± 0.77	38.54 ± 18.47
2015	6,562	1.00 (1.00–1.00) ^e^^f^	12.84 (12.07–13.76)^e^	56.30 (49.38–62.85)^e^^f^	1.69 (1.62–1.73)^e*f^	1.37 (1.31–1.50)^e^^f^	7.33 (6.87–7.95)^e*f^	36.00 (23.00–52.00)^e^^f^
		0.97 ± 0.74	12.97 ± 2.21	56.35 ± 10.81	1.67 ± 0.09	1.40 ± 0.13	7.41 ± 0.74	37.10 ± 20.59
2016	6,938	1.00 (0.00–1.00)^e^^f^^g^	13.08 (12.15–15.12)^e^^f^^g^	56.90 (49.88–63.90)^e^^f^^g^	1.70 (1.63–1.76)^e^^f^^g^	1.39 (1.32–1.52)^e^^f^^g^	7.45 (7.00–8.02)^e^^f^^g^	46.03 (37.83–52.05)^e^^f^^g^
		0.66 ± 0.71	13.49 ± 2.83	57.43 ± 11.16	1.70 ± 0.11	1.42 ± 0.13	7.50 ± 0.73	45.15 ± 10.99
2017	7,003	0.00 (0.00–1.00)^e^^f^^g^^h^	15.12 (12.39–17.76)^e^^f^^g^^h^	58.33 (51.16–67.13)^fgh^	1.73 (1.66–1.78)^e^^f^^g^^h^	1.36 (1.30–1.45)^e^^f^^g^^h^	7.16 (6.63–7.71)^e^^f^^g^^h^	35.59 (29.52–42.25)^e^^f^^h^
		0.55 ± 0.78	15.53 ± 4.24	59.07 ± 11.32	1.72 ± 0.10	1.39 ± 0.12	7.23 ± 0.73	36.21 ± 10.57
2018	5,365	1.00 (0.00–1.00)^e^^f^^g^^h^^i^	13.85 (12.22–17.42)^e^^f^^g^^h^^i^	62.58 (47.14–76.33)^e^^f^^g^^h^^i^	1.71 (1.67–1.75)^e^^f^^g^^h^^i^	1.35 (1.31–1.41)^e^^f^^g^^h^^i^	7.62 (7.22–7.93)^e^^f^^g^^h^^i^	32.51 (25.53–40.61)^e^^f^^g^^h^^i^
		0.87 ± 0.89	15.27 ± 4.41	62.90 ± 16.55	1.71 ± 0.15	1.37 ± 0.11	7.60 ± 0.56	33.64 ± 11.58
2019	7,180	1.00 (0.00–1.00)^e^^f^^g^^h^^i^^j^	16.52 (14.20–19.38)^e^^f^^g^^h^^i^^j^	58.20 (48.10–74.06)^e^^f^^g^^h^	1.72 (1.68–1.80)^e^^f^^g^^h^^i^^j^	1.40 (1.34–1.50)^e^^f^^g^^h^*^ij^	7.77 (7.31–8.06)^e^^f^^g^^h^^i^^j^	21.20 (16.21–28.16)^e^^f^^g^^h^^i^^j^
		0.76 ± 0.85	17.26 ± 3.89	60.93 ± 15.86	1.67 ± 0.35	1.42 ± 0.11	7.68 ± 0.78	23.38 ± 10.29
Total	46,951	1.00 (0.00–1.00)^***^	13.51 (12.34–16.00)^***^	58.69 (49.36–68.70)^***^	1.70 (1.65–1.75)^***^	1.40 (1.32–1.52)^***^	7.46 (6.99–7.97)^***^	35.00 (24.56–46.18)^***^
		0.79 ± 0.84	14.51 ± 3.54	59.48 ± 13.71	1.68 ± 0.16	1.43 ± 0.13	7.47 ± 0.74	35.50 ± 16.56

*Results were expressed as median (P_25_−P_75_); Kruskal-Wallis was used to compare differences in two and more groups*.

*The mean values and SDs were noted in the second row for each element*.

* *p < 0.05, compared with boys*.

** *p < 0.01, compared with boys*.

a *Compared with Group A, p < 0.01*,

b *compared with Group B, p < 0.01*,

c *compared with Group C, p < 0.01*,

^c^^*^ compared with Group C, p < 0.05.

e *Compared with Group 2013, p < 0.01*,

f *compared with Group 2014, p < 0.01*,

g *compared with Group 2015, p < 0.01*,

h *compared with Group 2016, p < 0.01*,

i *compared with Group 2017, p < 0.01*,

j *compared with Group 2018, p < 0.01*.

^e^^*^Compared with Group 2013, p < 0.05,

^h^^*^Compared with Group 2016, p < 0.05.

*** *p < 0.001*.

**p < 0.01.

Compared to the reference concentrations, we evaluated how many children had an overconcentration of Pb and Cd and how many had a deficient concentration of essential elements. The rates in different groups were compared by the chi-square test or Fisher's exact test as applicable. Finally, we used Spearman's rank correlation coefficient to evaluate the association of every pair of elements. The significance level was set at *p* < 0.05 (two-sided).

## Result

**Figure 1:**
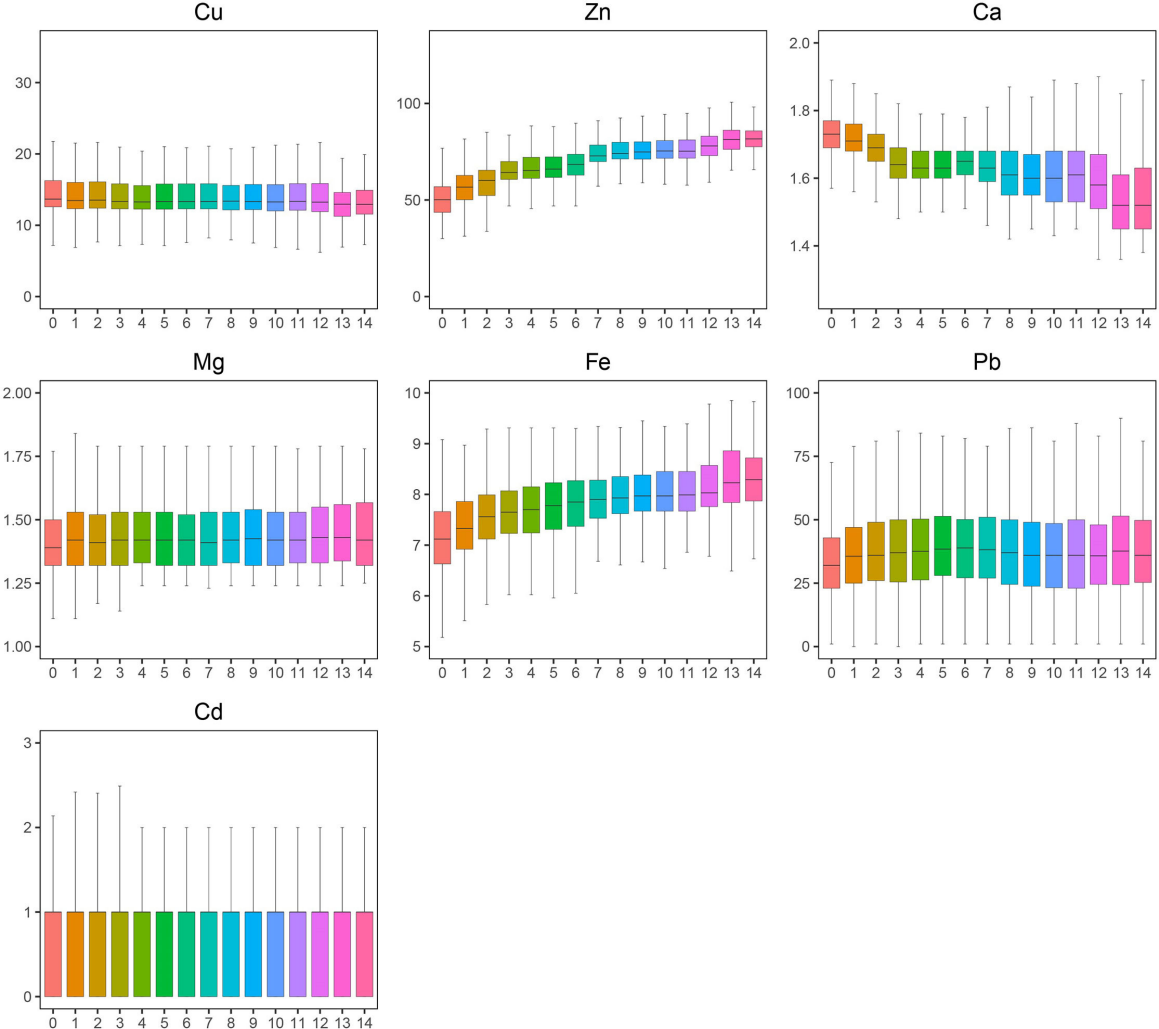
A trend of toxic and essential element levels in the blood from 0 to14 years old. The boxplot showed the median values of elements and their percentile ranges.

**Figure 2:**
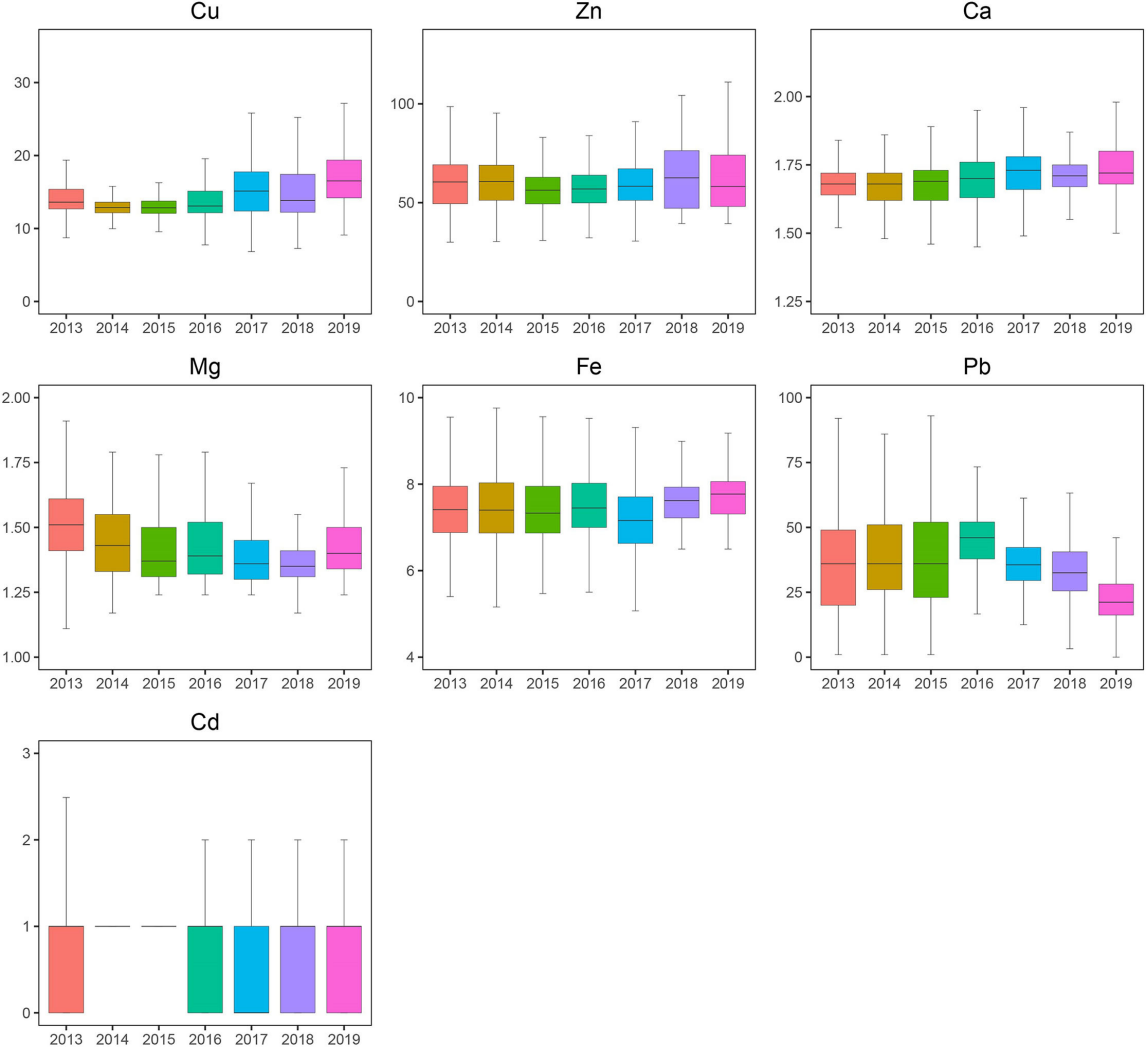
Blood levels of toxic and essential elements in the children aged 0~14 years by year, 2013–2019. The boxplot showed the median values of elements and their percentile ranges.

A total of 46,951 children aged 0–14 years were recruited for the study, and 55.67% of the subjects were boys. Significant differences were found in Ca, Pb, and Cd between boys and girls (Table 1), and girls had a higher level of Ca and lower levels of Pb and Cd than boys. Most elements changed very slightly and irregularly with age except for Zn and Fe, which showed an upward trend, and Ca, which showed a downward trend (Figure 1). After dividing the children into four age groups, we found that, when the child was older than 1 year, the element concentrations changed obviously because the B, C, and D groups of every element showed a significant difference from the A group of the same element. No difference was found among the other groups of Cd. However, Cu, Zn, Ca, Fe, and Pb showed significant differences in every two age groups, especially for Zn, Fe, and Ca with every *P* < 0.01; these three elements increased by 47.05%, increased by 11.80%, and decreased by 6.36%, respectively, as shown in Figure 1. Next, we evaluated the changes in elements by year from 2013 to 2019. Roughly, Cu and Ca showed an upward trend, Mg and Pb showed downward trends, and no obvious change was found in Fe, Zn, and Cd (Figure 2). Based on the data of Table 1, Pb showed the most apparent decrease of 41.11%, followed by Cu, which increased by 21.38%. Significant differences in Cd, Ca, and Mg in any 2 years could be found, even if the quartiles of Cd hardly changed, which might be explained by the fact that more than half of the participants in each year had Cd concentrations of 1.00 μg/L. Ca reached the highest point in 2017 with a concentration of 1.73 mmol/L and slightly decreased later but was generally higher than the levels before 2017. Mg showed a steady downward trend before 2018 but suddenly increased to 1.40 in 2019, which was lower than that in 2013 but higher than that in 2015.

Next, we calculated the overconcentration rate of the poisonous elements Pb and Cd and the deficiency rate of the other essential elements in different sex and age groups. Taking Pb <100 μg/L as the standard reference for Pb poisoning, 0.33% of boys and girls had higher concentrations of Pb (Table 2). In addition, blood Pb over 50 μg/L could be considered excessive Pb for children due to the critical period of childhood, and 20.19% of boys and 16.52% of girls met these criteria; the difference was significant. In addition, the boys showed a significantly higher Ca and lower Zn deficiency than the girls, and the former was consistent with Table 1. No significant sex differences were found in other groups. For the four age groups, the deficiencies in Fe, Zn, and Mg all showed a downward trend from Group A to Group D by 73.19, 97.83, and 100%, respectively. Meanwhile, Cu showed an upward trend with age by 87.05%. The largest change was in the deficiency of Ca, with a rate in Group D more than 19 times larger than that in Group A. Interestingly, the change in Ca was sudden after 6 years of age. A significant difference could be seen in any two groups in essential elements except for Mg. Regarding the toxic elements Pb and Cd, the children in Group B had a relatively higher rate of Pb > 150 μg/L and Cd > 2.5 μg/L, which was significantly different from Group A but not from Groups C or D. The children in Group C had a higher rate of Pb > 100 μg/L, Pb > 50 μg/L, and Cd > 5 μg/L, among which the first rate was significantly different from Group A and the second rate was significantly different from Groups A and B, but no significant difference was found between the last rate and any groups.

**Table 2:** The rates of elevated toxic elements and deficiency of essential elements in different age groups (%).

**Group**	***N***	**Pb > 150**	**Pb > 100**	**Pb > 50**	**Cd > 5**	**Cd > 2.5**	**Ca <1.55**	**Fe <7.52**	**Zn^#^ <58**	**Cu <11.8**	**Mg <1.12**
		**(μg/L)**	**(μg/L)**	**(μg/L)**	**(μg/L)**	**(μg/L)**	**(mmol/L)**	**(mmol/L)**	**(μmol/L)**	**(μmol/L)**	**(mmol/L)**
**Gender**
Boys	26,429	0.08	0.33	20.19	0.02	2.74	5.41	51.40	48.04	14.41	0.02
Girls	20,522	0.06	0.33	16.52^**^	0.01	2.82	4.96^**^	52.13	49.01^*^	14.01	0.02
**Age (years)**
A(0~)	19,262	0.04	0.13	11.91	0.01	2.46	1.10	69.50	79.16	10.66	0.04
B(1~)	12,803	0.10^a^^*^	0.40^a^	20.90^a^	0.02	3.21^a^	0.86^a*^	54.35^a^	52.35^a^	14.55^a^	0.02
C(3~)	6,056	0.07	0.53^a^	25.88^a^^b^	0.03	2.84	2.77^a^^b^	37.88^a^^b^	10.78^a^^b^	16.61^a^^b^	0
D(6~14)	8830	0.09	0.51^a^	24.78^a^^b^	0.00	2.77	22.19^a^^b^^c^	18.63^a^^b^^c^	1.72^a^^b^^c^	19.94^a^^b^^c^	0
Total	46,951	0.07	0.33^m^	18.59^m^	0.01	2.77^m^	5.22^m^	51.72^m^	48.46^m^	14.24^m^	0.02

# *Means Zn <58 μmol/L (0~0.99 years), 62 μmol/L (1~1.99 years), 66 μmol/L (2~2.99 years), 72 μmol/L (3~4.99 years), and 76.5 μmol/L (≥5 years) in accordance with the reference value in different age groups*.

*Results were expressed as percentage (%). Chi-square test or Fisher's exact test was used to compare differences between groups*.

** *p <0.01, compared with boys*.

* *p < 0.05, compared with boys*.

a *Compared with Group A, p < 0.01*,

^a^^*^compared with Group A, p < 0.05,

b *compared with Group B, p < 0.01*,

c *compared with Group C, p < 0.01*,

m *p < 0.01, between age groups*.

Finally, we used Spearman's rank correlation coefficient to evaluate the association of every pair of elements. Associations were found except for Zn and Cu, and Mg and Cu (Table 3). Some pairs, however, such as Cu and Cd, Zn and Cd, Mg and Cu and others with a *P* < 0.01, were also not considered associations because of the small correlation coefficient. Generally, the association was worth considering when the correlation coefficient was higher than 0.3, since other coefficients were relatively low or negligible. Based on this, Ca was positively correlated with Cu and negatively correlated with Zn, and Fe was positively correlated with Zn in this study.

**Table 3:** Correlation coefficient between different elements (r-value) (*n* = 46,951).

	**Cd**	**Cu**	**Zn**	**Ca**	**Mg**	**Fe**	**Pb**
Cd	1	−0.046^**^	0.011^*^	−0.068^**^	0.027^**^	0.042^**^	0.037^**^
Cu	-	1	0.007	0.357^**^	0.007	0.011^*^	−0.178^**^
Zn	-	-	1	−0.339^**^	0.099^**^	0.370^**^	0.061^**^
Ca	-	-	-	1	−0.076^**^	−0.265^**^	−0.154^**^
Mg	-	-	-	-	1	0.245^**^	0.022^**^
Fe	-	-	-	-	-	1	0.017^**^
Pb	-	-	-	-	-	-	1

* * p < 0.05*,

** *p < 0.01*.

## Discussion

Pb and Cd are found everywhere in daily life. In general, children come into contact with Pb in many ways, such as vehicle exhaust, factory fumes, environmental dust and paint, contaminated water and food, toys, and so on (29). In our study, the median BLL of children was 35.00 μg/L, ranging from 36.00 μg/L to 21.00 μg/L between 2013 and 2019 (Figure 1), which was lower than the previous reports conducted in the Chinese cities of Nanjing (41.16 μg/L) (30), Jinan (49.42 μg/L) (31), Beijing (42.8 μg/L) (32), Nanning (52.6 μg/L) (33), and Changchun (60.29 μg/L) (34) and slightly higher than the values reported in Sáo Paulo (Brazil, 2013) (31.2 μg/L) (35) and Wuhan in China (33.72 μg/L) (36). A total of 0.33% of all children had BLL > 100 μg/L, which was lower than that in Nanjing (1.3%) (30), Jinan (1.4%) (31), and Changchun (10%) (34). The low Pb and Pb poisoning rates can be explained by the following: (a) Most of the children in this study were younger than 3 years old (68.1%), and their range of activity was smaller than that of older children, reducing the opportunity for exposure to Pb. (b) Besides banning the use of leaded gasoline since 2000, the local government has attached great importance to the publicity and prevention of Pb exposure in children, such as placing restrictions on lead-contaminated paints and industrial emissions. The BLLs and Pb intoxication percentages (>50 μg/L) gradually increased with age, which was consistent with other studies (33–36). The boys had a higher mean BLL and a prevalence rate of Pb poisoning (BLL > 50 μg/L) than the girls, similar to other reports (33, 36, 37). Persistent efforts in reducing Pb exposure were needed for an average of 18.59% of all the children who had blood Pb levels >50 μg/L in this study.

Except for occupational exposure and smoking, food is the main source of Cd exposure for the general population (14). The objective of our study concentrated on children <3 years old (68.29%), especially children <1 year old (41.03%), whose staple food, we speculated, was breast milk or infant formulae. Cd concentrations in infant products were generally lower than those measured in most common food groups (38). In addition, baby food might be prepared more carefully by parents; thus, the concentration of Cd in our study was at a low level, and very few children had Cd poisoning; our results were lower than those reported by Zhao et al. (31) and Xu et al. (34).

Zn, Ca, Fe, Cu, and Mg are the most significant essential nutritional elements. Our data showed that the level of Ca gradually decreased with age, which was in accordance with the reports by Zhao (31) in Jinan. Overall, an average of 5.22% of children were Ca deficient, and Ca deficiency in older children was much higher than in younger children, with the prevalence of Ca deficiency from 1.10% in the 0 to 1 year age group to 22.19% in the 6–14 years age group. The change may have several possible explanations: (a) Calcium is the main component of bones and teeth. As bones develop, calcium gradually deposits on them; therefore, calcium in the serum declines with age. (b) Fine processing of grains and cereals results in large amounts of loss of minerals and vitamins, including Ca, in rice bran, (c) Lack of dairy products: milk intake decreases with age. Dairy is the main source of calcium, and the daily intake of dairy products in Chinese children is lower than the dietary recommendations of the Chinese Nutrition Society (39). (d) Vitamin D deficiency (40): Children might not have enough time to be exposed to ultraviolet rays; however, the duration of sunshine directly affects the conversion of vitamin D, and vitamin D deficiency reduces calcium absorption.

It is important to note that 48.46% of all children were Zn deficient, which was much more concerning than the data in the studies conducted by Kang-Sheng et al. (30). Ye et al. (36), and Zhao et al. (31). The overall median blood Fe level was 7.46 mmol/L, which was lower than the normal threshold of 7.52 mmol/L. Overall, Fe deficiency was 51.72%, lower than the data reported by Zhao et al. (31) and higher than data of the investigation conducted by Kang-Sheng et al. (30) and Ye et al. (36). Wu et al. (32) reported that there was no significant sex difference for Ca, Mg, and Fe. Chen et al. (33) concluded that the mean blood Pb and Cu levels of the boys were higher than those of the girls, but the mean blood Zn and Fe levels of the boys were lower than those of the girls. In our study, the blood levels of Fe in the boys were higher than those in the girls, which was inconsistent with the investigation conducted by Cao et al. (3) and Chen et al. (33). The prevalence of Zn and Fe deficiency tended to decrease with age. This phenomenon was consistent with previous studies in China (30, 31). There may be several explanations for this observation. First, children are in a period of rapid growth and development, and the body needs more Zn and Fe supplementation. Second, for young children whose supplementary food is not added in time, the staple food is breast milk or infant formulae or grains, and their gastrointestinal digestion and absorption capacity are relatively poor. Low biological utilization and absorption rates of Zn and Fe in grains and milk cannot meet the needs of infants, and infants who are fed milk or cereal for a long time are vulnerable to Zn and Fe deficiency. Third, with increasing age and the addition of food diversity, especially the increased intake of animal food, children's enhanced gastrointestinal digestion and absorption capacity will increase the intake of Fe and Zn. Meanwhile, unhealthy eating habits such as dietary bias and anorexia will cause Zn and Fe deficiency. In view of this point, greater attention is required in the supplementation of Zn and Fe in children's food.

Deficiencies in Ca, Fe, and Zn may enhance Pb absorption and promote Pb toxicity (18, 41). Studies on the correlation between Pb and other essential elements have been inconsistent. Chen et al. (33) noted that BLLs were positively correlated with blood Zn, Fe, and Mg levels and negatively correlated with blood Ca levels when children had BLLs <5 g/dl. Wu et al. (32) observed that there was a negative correlation of Pb with Zn, Mg, and Fe. Ahamed et al. (42) reported that there were significant negative correlations of blood Pb levels with Fe, Zn, and Ca. Positive correlations between Pb and Fe or Mg and a negative correlation between Pb and Ca were found by Li et al. (43). Positive correlations were found by Zhao et al. (31) when comparing Pb to Zn and Fe, and negative correlations of the blood levels of Pb with those of Cu, Mg, and Fe were observed by Ye et al. (36). In our study, blood Pb was positively correlated with Cd, Zn, Mg, and Fe but negatively correlated with Cu and Ca, while all the correlation coefficients were <0.3, which might not be sufficient to define their relevant relationship. The inconsistency in the correlation of these element concentrations may reflect the different metal levels in the local environment. Precise measurement of trace element levels in the environment may help solve this problem.

### Study Limitations

The limitation of our survey was that all the children we studied came for health examinations in the child health center of our hospital, so the children who did not come for physical check-ups were not recruited in this study. In addition, the same child may have more than one time undergone physical examination and element detection in the period of 2013–2019, especially for children whose element levels were above or below normal thresholds. In addition, one thing that must be noted is that total blood concentrations of essential trace elements are only one of the aspects used to evaluate child health but might not be a sufficiently good indicator of nutritional status. Many other indicators need to be considered when evaluating nutrient status (44). Nevertheless, the study's large sample size reduced its selection bias, and the database could provide a scientific basis for further intervention for local governments and hospitals.

## Conclusion

In this study, we evaluated the blood levels of essential and toxic elements in 46,951 children aged 0–14 years in Hunan, China, from 2013 to 2019. The levels of the essential elements Ca, Cu, and Mg and the toxic elements Pb and Cd were in the normal range in most children, whereas severe deficiencies in Zn and Fe were observed in the relatively younger children but improved with increasing age. Note that, even if children of different age groups have different levels of trace element deficiencies, their total blood concentrations might not be enough to be a good indicator of nutritional status. Therefore, supplementation with trace elements should be prudent. Although the prevalence of Pb intoxication in all children was not severe, the blood Pb concentration was at a relatively high level. We should make persistent efforts to reduce Pb exposure. To the best of our knowledge, this is the first large data analysis of elements in children in Hunan, China; hopefully, it provides a reference for the clinical guidance of child health.

## Data Availability Statement

The raw data supporting the conclusions of this article will be made available by the authors, without undue reservation.

## Ethics Statement

The studies involving human participants were reviewed and approved by the Ethics Committee of the Third Xiangya Hospital. Written informed consent from the participants' legal guardian/next of kin was not required to participate in this study in accordance with the national legislation and the institutional requirements. Written informed consent was not obtained from the minor(s)' legal guardian/next of kin for the publication of any potentially identifiable images or data included in this article.

## Author Contributions

ST and YYan: conceptualization, methodology, validation, formal analysis, investigation, and writing—original draft. LZ and ZY: investigation and methodology. HD: project administration. WH: methodology and investigation. MJ: investigation and resources. YYao and KH: software and validation. LL, PZ, and SX: validation. MZ and ZC: conceptualization, supervision, and validation. MY: writing—revised draft, supervision, and validation. All authors contributed to the article and approved the submitted version.

## Funding

This work was supported by research grants from the National Natural Science Foundation of China (No. 82070815).

## Conflict of Interest

The authors declare that the research was conducted in the absence of any commercial or financial relationships that could be construed as a potential conflict of interest.

## Publisher's Note

All claims expressed in this article are solely those of the authors and do not necessarily represent those of their affiliated organizations, or those of the publisher, the editors and the reviewers. Any product that may be evaluated in this article, or claim that may be made by its manufacturer, is not guaranteed or endorsed by the publisher.
